# Investigation of breast cancer microstructure and microvasculature from time-dependent DWI and CEST in correlation with histological biomarkers

**DOI:** 10.1038/s41598-022-10081-7

**Published:** 2022-04-20

**Authors:** Yuko Someya, Mami Iima, Hirohiko Imai, Akihiko Yoshizawa, Masako Kataoka, Hiroyoshi Isoda, Denis Le Bihan, Yuji Nakamoto

**Affiliations:** 1 Department of Diagnostic Imaging and Nuclear Medicine, Graduate School of Medicine, Kyoto University, Kyoto, 606-8507 Japan; 2 Department of Clinical Innovative Medicine, Institute for Advancement of Clinical and Translational Science, Kyoto University Hospital, Kyoto, 606-8507 Japan; 3 Department of Systems Science, Graduate School of Informatics, Kyoto University, Kyoto, 606-8501 Japan; 4 Department of Diagnostic Pathology, Kyoto University Hospital, Kyoto, 606-8507 Japan; 5 NeuroSpin/Joliot, CEA-Saclay Center, Paris-Saclay University, 91191 Gif-sur-Yvette, France; 6 Human Brain Research Center, Kyoto University Graduate School of Medicine, Kyoto, 606-8507 Japan; 7 National Institute for Physiological Sciences, Okazaki, 444-8585 Japan

**Keywords:** Cancer, Oncology

## Abstract

We investigated the associations of time-dependent DWI, non-Gaussian DWI, and CEST parameters with histological biomarkers in a breast cancer xenograft model. 22 xenograft mice (7 MCF-7 and 15 MDA-MB-231) were scanned at 4 diffusion times [T_d_ = 2.5/5 ms with 11 b-values (0–600 s/mm^2^) and T_d_ = 9/27.6 ms with 17 b-values (0–3000 s/mm^2^), respectively]. The apparent diffusion coefficient (ADC) was estimated using 2 b-values in different combinations (ADC^0–600^ using b = 0 and 600 s/mm^2^ and shifted ADC [sADC^200–1500^] using b = 200 and 1500 s/mm^2^) at each of those diffusion times. Then the change (Δ) in ADC/sADC between diffusion times was evaluated. Non-Gaussian diffusion and intravoxel incoherent motion (IVIM) parameters (ADC_0_, the virtual ADC at b = 0; K, Kurtosis from non-Gaussian diffusion; *f*, the IVIM perfusion fraction) were estimated. CEST images were acquired and the amide proton transfer signal intensity (APT SI) were measured. The ΔsADC_9–27.6_ (between ${\text{sADC}}_{{9\,{\text{ms}}}}^{200{-}1500}$ and ${\text{sADC}}_{{27.6\,{\text{ms}}}}^{200{-}1500}$ and ΔADC_2.5__sADC_27.6_ (between ${\text{ADC}}_{{2.5\, {\text{ms}}}}^{0{-}600}$ and ${\text{sADC}}_{{27.6\,{\text{ms}}}}^{200{-}1500}$) was significantly larger for MCF-7 groups, and ΔADC_2.5__sADC_27.6_ was positively correlated with Ki67_max_ and APT SI. ADC_0_ decreased significantly in MDA-MB-231 group and K increased significantly with T_d_ in MCF-7 group. APT SI and cellular area had a moderately strong positive correlation in MDA-MB-231 and MCF-7 tumors combined, and there was a positive correlation in MDA-MB-231 tumors. There was a significant negative correlation between APT SI and the Ki-67-positive ratio in MDA-MB-231 tumors and when combined with MCF-7 tumors. The associations of ΔADC_2.5__sADC_27.6_ and API SI with Ki-67 parameters indicate that the T_d_-dependent DW and CEST parameters are useful to predict the histological markers of breast cancers.

## Introduction

MR imaging can provide anatomical and functional information about both normal tissues and diseased ones, such as tumors^1^. Among the quantitative MRI methods available, diffusion-weighted imaging (DWI) and chemical exchange saturation transfer (CEST) imaging might be useful to reveal the microstructure and microvasculature within cancer lesions^1–3^. Although histological assessment from biopsy remains the basis for determining treatment, many cancers are very heterogeneous, and biopsy often fails to capture their heterogeneity, as the amount of the tumor obtained with biopsy is very limited. Whole tumor mapping using MRI might provide additional information about the correlations between heterogeneity and histology, which could be useful for evaluating the extent of tumors in the preoperative state or for decision making about neoadjuvant cancer treatment. Regarding breast tumors, which are the leading cause of cancer death among women worldwide^4^, imaging biomarkers with greater specificity to tumor biology like proliferation activity have been desired to facilitate selection of treatment options^5^.

Apparent diffusion coefficient (ADC) values obtained from DW images have been widely used in oncologic imaging. In various tumors, ADC has been recognized as a sensitive surrogate for elevated cellularity, contributing to reduced motion of water^6^. However, the association of ADC values with tumor proliferative markers, such as Ki-67 expression, in breast cancer is still controversial^7,8^. Lately, the diffusion time (T_d_) has been identified as an important acquisition parameter that can influence ADC values^9^. Recent investigations have emphasized the importance of T_d_ because diffusion is hindered by many obstacles in biological tissues, decreasing the level of water molecule displacement compared with free diffusion paths. The ADC value decreases when T_d_ gets longer, as water molecules have a greater probability of interacting with microscopic tissue features such as cell membranes and fibrous tissues. Several studies have reported that various types of malignant tumors revealed the decrease in ADC values with long T_d_^10–13^.

There have been explorations of several non-Gaussian DWI and intravoxel incoherent motion (IVIM) parameters that are useful for the differentiation of malignant and benign breast lesions^14–17^. Non-Gaussian DWI, which can be investigated using multiple b values (including high b values), is more sensitive to water diffusion hindrance by tissue constitutive elements (e.g., cell membranes, fibers). For breast cancer, a positive association between kurtosis and Ki-67 expression has been shown^18^. In contrast, IVIM MR imaging reflects both the diffusion of the water molecules and the perfusion (i.e., pseudo-random flow of blood in capillary networks). Perfusion-related parameters, including the pseudo-diffusion coefficient (D*) and the perfusion fraction (*f*), may predict tumor microvasculature. Regarding tumor angiogenesis, a correlation between microvessel density (investigated by CD31 staining) and IVIM parameters has been observed in various types of cancers^19–21^.

In this study we have also used a “shifted ADC” (sADC) which is calculated using 2 key b values (here 200 and 1500 s/mm^2^) to enhance a combined sensitivity to both diffusion and non-Gaussian diffusion, as well as IVIM effects^22^.

CEST has been increasingly considered as a promising molecular imaging method that can evaluate the presence of low concentrations of molecules other than water^23^. CEST might provide complementary molecular information related to cancer metabolism^24^, and its utility has been explored in oncology, including breast cancer xenograft^25,26^ and human breast cancer^27–29^ studies. Amide protons from mobile proteins and peptides are typical endogenous CEST agents. Several investigations have reported that amide proton transfer (APT) imaging might be useful to predict tumor proliferation indices such as the Ki-67 index^29–32^.

Our purpose was to investigate the associations of time-dependent DWI, non-Gaussian DWI, IVIM, and CEST parameters obtained from 7 T MRI of a murine breast xenograft model with histological biomarkers.

## Results

### Time-dependent DWI and CEST

**Figure 1:**
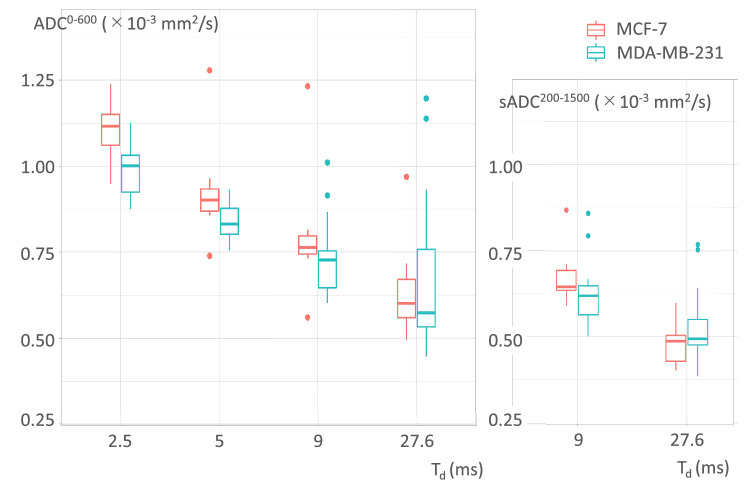
Box-whisker plots of ADC^0–600^ and shifted ADC^200–1500^ (sADC^200–1500^) against diffusion time.

**Figure 2:**
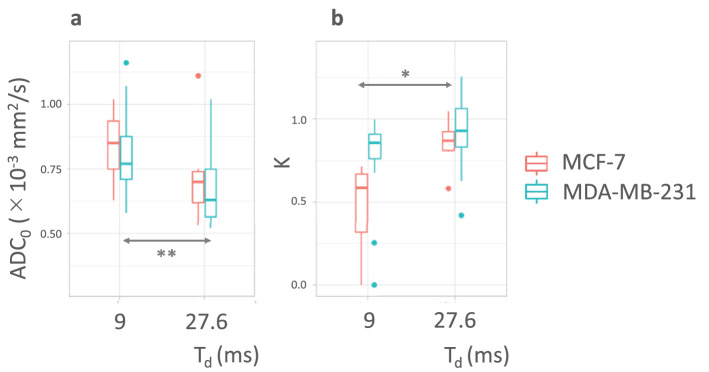
Box-whisker plots of (**a**) ADC_0_ and (**b**) K parameters against diffusion time. *P < 0.05, **P < 0.01. ADC_0_ in the MDA-MB-231 group decreased significantly (*P* < 0.01), and ADC_0_ in MCF-7 group decreased (*P* = 0.16), with increased diffusion time. K in the MCF-7 group increased significantly (*P* < 0.05) and K in the MDA-MB-231 group increased (*P* = 0.06) with increased diffusion time.

The DWI parameters, which were dependent on T_d_ in the two tumor xenograft models, are provided in Table 1. The T_d_ dependence of the DWI parameters is shown in Figs. 1 and 2. Both ADC^0–600^ and sADC^200–1500^ exhibited a tendency to decrease when the T_d_ value increased (Fig. 1). The superscripted part of ADC/sADC indicates the b-values and the subscripted part of those indicates the diffusion time, T_d_.

**Table 1:** MRI parameters for the MCF-7 and MDA-MB-231 groups. *P* < 0.05*, *P* < 0.01**, considered as statistically significant.

	T_d_ (ms)	MCF-7	MDA-MB-231	*P*-value
**DWI parameters**		**N = 7**	**N = 15**	
ADC^0–600^ (× 10^–3^ mm^2^/s)	2.5	1.10 ± 0.09	0.99 ± 0.08	0.011*
	5	0.93 ± 0.17	0.84 ± 0.06	0.091
	9	0.81 ± 0.21	0.79 ± 0.23	0.448
	27.6	0.65 ± 0.16	0.68 ± 0.23	0.837
ΔADC^0–600^ (%) between 2.5 and 27.6 ms		41.5 ± 12.7	31.3 ± 21.0	0.210
sADC^200–1500^ (× 10^–3^ mm^2^/s)	9	0.68 ± 0.09	0.62 ± 0.10	0.066
	27.6	0.48 ± 0.07	0.53 ± 0.12	0.490
ΔsADC^200–1500^ (%) between 9 and 27.6 ms		29.6 ± 4.4	14.6 ± 15.8	0.002**
ΔADC_2.5__sADC_27.6_ (%) between ADC_2.5 ms_ and sADC_27.6 ms_		56.7 ± 4.4	46.4 ± 12.4	0.026*
**Non-Gaussian diffusion parameters**		**N = 7**	**N = 15**	
ADC_0_ (× 10^–3^ mm^2^/s)	9	0.84 ± 0.15	0.82 ± 0.17	0.546
	27.6	0.72 ± 0.19	0.68 ± 0.16	0.546
K	9	0.47 ± 0.27	0.76 ± 0.28	0.005**
	27.6	0.85 ± 0.15	0.92 ± 0.22	0.332
**IVIM parameters**		**N = 7**	**N = 15**	
*f* (%)	9	6.7 ± 5.6	5.8 ± 4.8	0.582
	27.6	7.9 ± 5.6	5.2 ± 5.6	0.267
D* (× 10^–3^ mm^2^/s)	9	7.11 ± 3.59	6.10 ± 1.89	0.891
	27.6	5.70 ± 2.02	5.73 ± 2.37	0.989
**CEST parameters**		**N = 5**	**N = 12**	
APT SI (%)		6.48 ± 3.59	3.58 ± 1.91	0.113

The ${\text{ADC}}_{{2.5\,{\text{ms}}}}^{0{-}600}, {\text{ADC}}_{{5\,{\text{ms}}}}^{0{-}600}, and \,\,{\text{ADC}}_{{9\,{\text{ms}}}}^{0{-}600}$values at T_d_ = 2.5, 5, and 9 ms, and ${\text{sADC}}_{{9\,{\text{ms}}}}^{200{-}1500}$ values at T_d_ = 9 ms were lower in the MDA-MB-231 group than the MCF-7 group, whereas the ${\text{ADC}}_{{27.6\,{\text{ms}}}}^{0{-}600}$ and ${\text{sADC}}_{{27.6\,{\text{ms}}}}^{200{-}1500}$ values at T_d_ = 27.6 ms were lower in the MCF-7 group than MDA-MB-231 group. The ΔsADC_9–27.6_ and the ΔADC_2.5__sADC_27.6_ in the MCF-7 group were significantly larger than those in the MDA-MB-231 group (29.6 ± 4.4% vs. 14.6 ± 15.8%, *P* < 0.01; 56.7 ± 4.4% vs. 46.4 ± 12.4%, *P* < 0.05; respectively). The ΔADC_2.5–27.6_ had no significant difference between two xenograft models (41.5 ± 12.7% in the MCF-7 group vs. 31.3 ± 21.0% in the MDA-MB-231 group; *P* = 0.21).

Non-Gaussian diffusion parameters (ADC_0_, the virtual ADC at b = 0 s/mm^2^; K, Kurtosis from non-Gaussian diffusion) were estimated at T_d_ = 9 ms and 27.6ms. The MDA-MB-231 group had a significantly higher K value than the MCF-7 group at T_d _= 9 ms (0.47 ± 0.27 vs. 0.76 ± 0.28, *P *< 0.01). However, K had no significant T_d_ dependence in either xenograft model. There was no significant difference in any other IVIM or non-Gaussian diffusion parameters between the two different types of tumor xenografts (Table 1). In the MDA-MB-231 group, the ADC_0_ value was significantly lower with increased T_d_ (*P *< 0.01) and, in the MCF-7 group, the ADC_0_ value insignificantly decreased with increased T_d_ (*P *= 0.16). The K value was significantly higher in the MCF-7 group (*P *< 0.05) with increased T_d_, and K in the MDA-MB-231 group insignificantly increased (*P *= 0.06) with increased T_d_ (Fig. 2).

**Figure 3:**
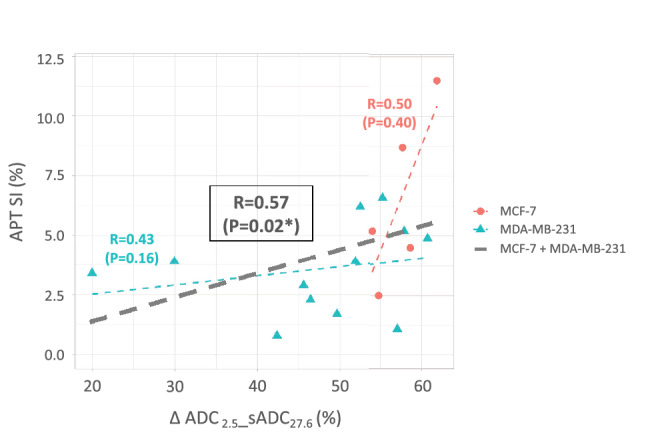
Scatter plots of ΔADC_2.5__sADC_27.6_ against APT SI. The Spearman’s rank correlation coefficients R, the corresponding *P* values in each tumors are shown in the figure. The black dashed line indicates the relationship between ΔADC_2.5__sADC_27.6_ and APT SI in the entire tumors (mixing MCF-7 and MDA-MB-231 groups).

We conducted CEST imaging on 5 and 12 mice with MCF-7 and MDA-MB-231 tumors, respectively. APT SI was defined as the MTR_asym_ value at 3.5 ppm. There was no significant difference of APT SI between the two different types of tumor xenografts, as shown in Table 1. APT SI had a significant positive correlation with ΔADC_2.5__sADC_27.6_ (R = 0.57, *P *< 0.05) in mixing MDA-MB-231 and MCF-7 tumors (Fig. 3). The ΔsADC_9–27.6_ had a tendency to correlate positively with APT SI, but not significantly (R = 0.48, *P* = 0.052). The ΔADC_2.5–27.6_ had no correlation with APT SI (R = 0.20, *P* = 0.45).

### Association between MRI (DWI and CEST) parameters and histological biomarkers

**Figure 4:**
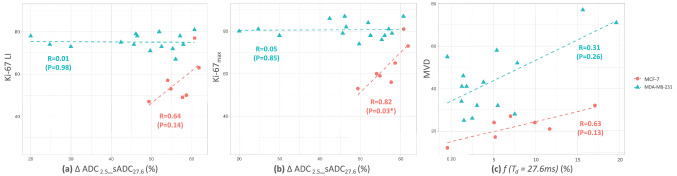
Scatter plots of (**a**) ΔADC_2.5__sADC_27.6_ against Ki-67 LI, (**b**) ΔADC_2.5__sADC_27.6_ against Ki-67_max_, and (**c**) *f* (T_d_ = 27.6 ms) against MVD. The Ki-67 indices in the MDA-MB-231 tumors revealed abnormally high expression, as shown in (**a**) and (**b**).

The association between DWI parameters and histological biomarkers is summarized in Fig. 4. In the MCF-7 group, the ΔADC_2.5__sADC_27.6_ had a positive correlation with Ki-67 LI, but the correlation was not significant (R = 0.64, *P *= 0.14, Fig. 4a). There was a positive correlation between the ΔADC_2.5__sADC_27.6_ and Ki-67_max_ (R = 0.82, *P *< 0.05, Fig. 4b). In the MDA-MB-231 group, the Ki-67 index revealed abnormally high expression and had a small standard deviation (SD). Hence, there was no correlation between the ΔADC_2.5__sADC_27.6_ and the Ki-67 tumor proliferation index. Each ADC value using a different T_d_ had no significant correlation with the Ki-67 index. There was no significant association between MVD and *f* with T_d_ values of either 9 or 27.6 ms. However, at T_d_ = 27.6 ms, a weak trend was observed between them (R = 0.63, *P* = 0.13 for MCF-7 and R = 0.31, *P* = 0.26 for MDA-MB-231).

**Figure 5:**
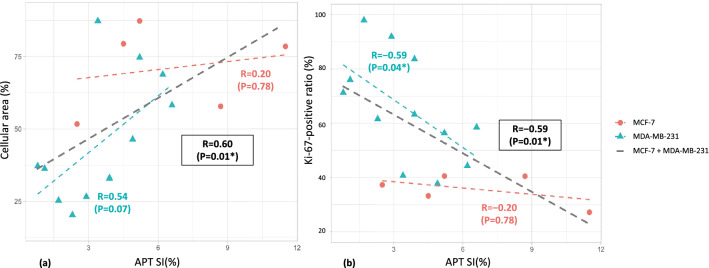
Scatter plots of (**a**) APT SI against cellular area, (**b**) APT SI against Ki-67-positive ratio. The Spearman’s rank correlation coefficients R, the corresponding *P* values in each tumor are shown in the figure. The black dashed line indicates the relationship between APT SI and histological biomarkers in the entire tumors (mixing MCF-7 and MDA-MB-231 groups).

The relationships between CEST parameters and histological biomarkers are summarized in Fig. 5. A positive correlation between APT SI and cellular area was shown in MDA-MB-231 tumors and in MDA-MB-231 and MCF-7 tumors combined (R = 0.54, *P* = 0.07 and R = 0.60, *P* = 0.01, respectively). APT SI had no significant correlation with the Ki-67 index. APT SI had a significant negative correlation with the Ki-67-positive ratio in MDA-MB-231 tumors and in MDA-MB-231 and MCF-7 tumors combined (R = − 0.59,* P* = 0.04 and R = −0.59, *P* = 0.01, respectively).

**Figure 6:**
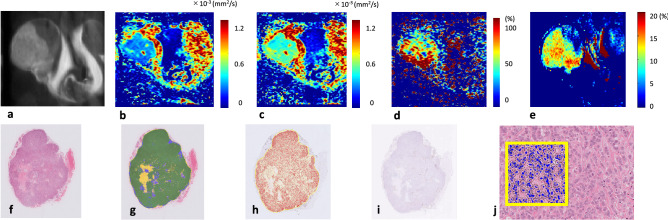
Representative case of MCF-7 tumor. MR images: (**a**) T2WI, (**b**) sADC map (diffusion time, T_d_ = 9 ms), (**c**) sADC map (T_d_ = 27.6 ms), (**d**) ΔsADC^200–1500^ map, (**e**) APT imaging map, and whole tumor histological images: (**f**) H&E staining, (**g**) cellular area map, (**h**) Ki-67-positive cell map, (**i**) CD31 staining. (**j**) H&E staining on high power magnification (× 400). Yellow line squares with 200 μm sides were drawn on (**j**). The decrease of ADC with T_d_ is evident. The dorsal part of the tumor shows high ΔsADC^200–1500^ and APT SI.

**Figure 7:**
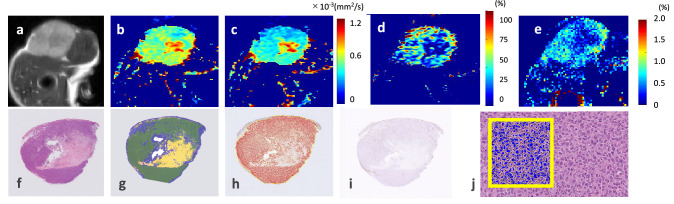
Representative case of MDA-MB-231 tumor. MR images: (**a**) T2WI, (**b**) sADC map (diffusion time, T_d_ = 9 ms), (**c**) sADC map (T_d_ = 27.6 ms), (**d**) ΔsADC^200–1500^ map, (**e**) APT imaging map, and whole tumor histological images: (**f**) H&E staining, (**g**) cellular area map, (**h**) Ki-67-positive cell map, (**i**) CD31 staining. (**j**) H&E staining on high power magnification (× 400). Yellow line squares with 200 μm sides were drawn on (**j**). The decrease of ADC with T_d_ is evident. Central necrosis is apparent in regions with low ΔsADC^200–1500^ and APT SI.

Figures 6 and 7 show representative whole tumor histological images (H&E staining, cellular area map, Ki-67-positive cell map, and CD31 staining), MR images (T2WI, sADC_9ms_ map, sADC_27.6ms_ map, the ΔsADC_9–27.6_ map, and APT imaging map), and H&E staining on high power magnification. Large ΔsADC_9–27.6_ and high APT SI were evident in the viable tumor regions.

### Histopathologic features

The histopathological features of MDA-MB-231 and MCF-7 xenograft models are summarized in Supplementary Table S1 online. The cell size of the MDA-MB-231 tumors was smaller than that of the MCF-7 group (12.2 ± 0.3 μm vs. 15.2 ± 0.7 μm, *P *< 0.05), and the cellular area of the MDA-MB-231 group was lower than that of the MCF-7 group (51.3% ± 22.7% vs. 71.8% ± 12.2%, *P* < 0.05). In immunohistochemical evaluation, Ki-67 LI, Ki-67_max_, and MVD were significantly higher in the MDA-MB-231 group than the MCF-7 group (75.5% ± 3.3% vs. 58.8% ± 11.5%, 80.7% ± 3.9% vs. 63.9% ± 10.0%, and 44.1 ± 15.9 vs. 23.5 ± 6.8, respectively; *P* < 0.01). The Ki-67-positive ratio, Ki-67 positive area divided by cellular area, and indices using whole-slide imaging were also significantly higher in the MDA-MB-231 group than the MCF-7 group (60.2% ± 20.4% vs. 41.5% ± 12.5%, *P* < 0.01). A strong positive correlation between cellular area and Ki-67 positive area was found in the MDA-MB-231 group (R = 0.74, *P* < 0.01), but not in the MCF-7 group (R = 0.41, *P* = 0.32).

## Discussion

In this study, we investigated the association between DW parameters measured at different T_d_ values (including ADC, sADC, IVIM, and non-Gaussian DWI), CEST parameters, and histopathological features, especially tumor proliferative markers, using murine xenograft models of human breast cancer. We found that the ΔADC_2.5__sADC_27.6_ using different T_d_ values had a significant positive correlation with APT SI. Previous studies have reported that time-dependent DWI or CEST imaging might provide information about tumor microstructure indicating tumor proliferation in oncologic imaging^29–33^. Comparisons of APT SI and IVIM parameters in cervical cancers^34^, gliomas^35^, and hepatocellular carcinomas^36^, as well as comparisons of APT SI and diffusion kurtosis imaging in rectal carcinomas^37^, have been reported. However, the relationship between time-dependent DWI parameters, as tissue level markers, and CEST parameters, as molecular level markers, is still unknown, to the best of our knowledge.

We used two different human breast cancer cell lines: an estrogen-dependent tumor cell line (MCF-7) and an aggressive, triple negative breast tumor cell line (MDA-MB-231). The MDA-MB-231 tumor cells showed smaller cell sizes, larger necrotic areas, higher Ki-67 expression, and greater MVD, suggesting histopathological malignancy, which is consistent with previous studies^38–42^. Although these histopathological features appear very different, single measurements of diffusion, IVIM, and CEST quantitative parameters were not useful to distinguish between the two different tumor types. Only ${\text{ADC}}_{{2.5\,{\text{ms}}}}^{0{-}600}$ values at the shortest T_d_ (2.5 ms using oscillating gradient spin-echo (OGSE)), ΔsADC_9–27.6_, ΔADC_2.5__sADC_27.6_ and K values at T_d_ = 9 ms showed statistically significant differentiation between the two xenograft groups.

The observed decrease in ADC and sADC values with T_d_ in breast tumors was in agreement with the literature, as were the results of our previous investigation^11,33,43^. This confirms the hypothesis that diffusion hindrance in tumors increases when the T_d_ value increases, as more molecules might hit the increased number of obstacles such as cell membranes. Our results also confirm that ADC value is a T_d_-dependent quantitative marker and thus that standardization of acquisition parameters in diffusion MRI is important, especially for multicenter studies^44^. Indeed, although the T_d_ settings available on clinical MRI scanners used to be similar, the availability of new, stronger gradient hardware now allows access to a broad range of T_d_ values while maintaining high b values. In general, the previous studies implicated that the ADC values obtained at very short T_d_ might diminish the contrast for differentiation between lesion types (i.e., malignant vs. benign lesions) because of a reduced sensitivity to diffusion hindrance ^11,33^. Our study revealed that only ${\text{ADC}}_{{2.5\,{\text{ms}}}}^{0{-}600}$ values at the shortest T_d_ (2.5 ms using OGSE) can distinguish between two xenograft models. These results might suggest that the ADC obtained at short T_d_ values could capture the movement of water molecules in all tissue compartments on the cellular and subcellular scales. In this study, we used a tumor model that mixed cellular and necrotic components in various proportions and ROIs on the whole tumor. The variation of ADC values is particularly large in the MDA-MB-231 group with a large necrotic area. As the T_d_ is increased, the water molecule displacement becomes larger, and then ADC values at long T_d_ values may indicate an increase in SD, reflecting the heterogeneity of the tissue. We considered that the ADC values at T_d_ = 2.5 ms, which can characterize the diffusion motion on a small scale, captures the small cell size (many cell membranes) of the MDA-MB-231 tumors, resulting in significantly lower ADC values in the MDA-MB-231 groups. These results also suggest that setting an appropriate T_d_ is troubling, however the change in ADC values measured at multiple T_d_ (ΔADC or ΔsADC) might be a new MRI parameter. These relatively easy-to-calculate values suggest the possibility of obtaining additional information about the structure of microtissues, such as cell size, cell density, or cell membrane^45^.

A previous study that compared between benign and malignant tumors revealed greater ADC change in malignant than benign lesions^33,43^. In this study, we assumed that more malignant pathological features like active proliferation, tight structure, and small cell spacing hinder the diffusion of water molecules inside tissue and ultimately decrease the ADC value in MDA-MB-231 tumors. However, in this study, the more aggressive MDA-MB-231 tumors had less ΔADC_2.5–27.6_, ΔsADC_9–27.6_ and ΔADC_2.5__sADC_27.6_ than the MCF-7 tumors. The presence of a larger necrotic area because of MDA-MB-231’s aggressiveness is assumed to be one of the causes of the lower ΔADC or ΔsADC observed in those tumors. There should be almost no structure in necrotic areas, which means that diffusion should be less hindered and almost free. Indeed, the areas with small ΔsADC_9–27.6_ corresponded to the necrotic areas in some tumors, as shown in Figs. 6 and 7. Investigation of T_d_’s dependence on tumor characterization provides new insight to supplement standard ADC values, and maps of ADC changes were useful for highlighting tumor features such as heterogeneity, as shown in this study. Furthermore, our result that the ΔADC^0–600^, calculated using standard ADC values with b values of 0 and 600 s/mm^2^, were not able to distinguish between two different xenografts is an interesting point. The ΔsADC_9–27.6_, excluding the signal of b value of 0 and using relatively high b value, is suggested to reflect non-Gaussian diffusion effect and to be more useful to estimate the tumor microstructure, because the behavior of DW signals is non-Gaussian, especially in highly restricted tissues such as cancers with cell proliferation.

A strong positive correlation between Ki-67_max_ and ΔADC_2.5__sADC_27.6_ was found in MCF-7 tumors (R = 0.82, *P* < 0.05). Cells with high Ki-67 expression tend to have high cellular proliferation and tight tissue structure, which hinders diffusion of water molecules inside and between these cells. No statistically significant correlation between tumor proliferative markers and ΔADC_2.5__sADC_27.6_ was found in MDA-MB-231 tumors. The MDA-MB-231 cell line has been known to express very high levels of Ki-67: almost 100% of these cells stain positively for Ki-67^38^, in contrast to human breast cancer. We also found in our study that MDA-MB-231 cell lines had abnormal expression and a small standard deviation of Ki-67 LI (75.5% ± 3.3%), so caution is required when extrapolating results obtained with xenograft models to clinical cancer when considering the association between the Ki-67 marker and MR parameters. Indeed, our single measurement of the ADC value for each T_d_ value had no correlation with any tumor proliferative biomarkers. It has often been found that ADC values are linked to both cell density and proliferation rate (measured by Ki-67) in tumors^46,47^. However, ADC can also be affected by other tissue characteristics, such as vascularity and extracellular water diffusivity. The rate of change of ADC with T_d_ appears to be more promising than the ADC value itself for highlighting differences in the tumor environment. Furthermore, the differences between ADC values observed in various tissue types at different T_d_ values might reflect functional differences in diffusion hindrance (e.g., related to membrane permeability to water^45^) in addition to microstructure (related to cell geometry)^48,49^.

There was a tendency of ADC_0_ decrease and K increase when increasing T_d_ from 9 to 27.6 ms in both xenograft models, further confirming the increase in diffusion hindrance. No significant differences of *f*, D*, or the time dependence of IVIM parameters were found between the two tumor groups. The differences between various IVIM models (the exponential model and sinc model)^50,51^ might explain some time-dependence of IVIM parameters. In this study, we used an exponential model that is valid as long as T_d_ is sufficiently long, but there is a general consensus that this model might not be accurate at small time scales, explaining the moderate correlation between *f* and MVD found only at long T_d_ (27.6 ms) and not at short T_d_. IVIM MRI might provide quantitative parameters of angiogenesis, like *f*, which should increase with the proliferation of neovascularity in some tumors. Tumor angiogenesis is essential for tumor growth and metastasis, and the establishment of a noninvasive imaging tool is essential to obtain the degree of angiogenesis noninvasively. As *f* is expected to be useful as an indicator of the degree of angiogenesis, further investigation with a larger group of subjects is desirable.

There was no significant association between APT SI and Ki-67 LI in our study. One reason for this result might be the abnormally high rate of Ki-67-positive cells, which was found especially in the MDA-MB-231 model. Second, Ki-67 LI was calculated as the mean positive cell count in the cellular area, and it therefore could not be directly compared with the mean APT SI value, which was calculated from the ROI covering the whole tumor including necrosis. Although several previous studies have reported positive correlations between APT SI and Ki-67 LI in gliomas^30^, meningiomas^31^, and rectal carcinomas^32^, no correlation was found in rectal adenocarcinomas^52^. There have been few studies on the utility of APT imaging in the differential diagnosis of different breast tumors^27,29^, and further investigation will be required to determine whether there is an association between APT SI and Ki-67 LI.

The positive correlation found between cellular area and APT SI (as shown in Fig. 5) is consistent with the general view that mobile protons in the cytoplasm (and mobile proteins and peptides in tumors^53^) are the major sources of APT signals. Malignant tumors with high Ki-67 expression have higher cellularity and increased cytosolic protein in the cytoplasm, resulting in higher APT SI^3^. In contrast, in our study, a negative association was observed between the Ki-67 positive ratio and APT SI (Fig. 5). The motivations for establishing the Ki-67 positive ratio (i.e. the ratio of Ki-67-positive area divided by the cellular area) were to reduce bias in ROI selection and to analyze the value equivalent to the conventional Ki-67LI in ROIs covering the whole tumor using Halo. Because Ki-67 is usually localized to the nucleus when detected by immunohistochemistry (an unusual cell membrane and cytoplasmic pattern of Ki-67 reactivity has been rarely described^54^, a large Ki-67-positive ratio means a small cytoplasmic area. In Jiang’s report^55^, primary central nervous system lymphomas (PCNSL) had significantly lower APT SI than high-grade gliomas, and this finding was hypothetically attributed to higher nucleus–cytoplasm ratios (N/C) in PCNSLs, which would reduce the amount of cytoplasmic protein. No significant correlation between APT and the Ki-67 positive ratio was observed in MCF-7 cells, which have a small N/C and varying Ki-67 positive cell rates. The decreased APT SI signal in MDA-MB-231 tumors may have been caused by high N/C, but this interpretation is not straightforward.

A moderate positive correlation between ΔADC_2.5__sADC_27.6_ and APT SI was found in our study, suggesting a link between tumor microstructure at the cellular scale and molecular status. Both parameters have been reported to reflect the tumor proliferative potential, as shown by Ki-67 expression, reflecting pathological malignancy, although diffusion MRI and CEST rely on totally different mechanisms. The ΔsADC_9–27.6_ maps and APT maps (Figs. 5 and 6) look very similar, including a signal decrease in the necrotic area. Because the standard ADC and shifted ADC values calculated using 2 b values did not correlate significantly with APT SI, ADC change using multiple T_d_ values may be useful as additional information to conventional DW parameters for distinguishing and evaluating two different types of malignancies, as in the present study.

The limitations of this study include that the number of mice was relatively small and that xenograft models were used. Xenograft models provide a whole organism environment for tumor growth, but using immunocompromised mice and differences associated with the implantation site might be limitations. For example, the observed aberrant expression of Ki-67 is different from that in human breast cancer. Thus, we cannot directly extrapolate our results to humans. Further investigation is warranted to verify the associations of DWI and CEST parameters with histopathological biomarkers. Then, in this study, we are not able to compare simply between ΔADC_2.5–27.6_ and ΔsADC_9–27.6_ because the using T_d_ to calculate these change values were different. The maximal b value in OGSE was 600 s/mm^2^ for maintaining the same TE as those in PGSE, hence the ΔsADC_9–27.6_ (high key b value of 1500 s/mm^2^) was calculated at T_d_ = 9 ms and 27.6 ms. Furthermore, we should note that keeping the TE constant between measurements of different T_d_ is essential to clearly attribute changes in the measured quantities to the time-dependent diffusion properties, since different T2 relaxation properties may change the signal fraction in different compartments. Furthermore, the CEST effect observed at the frequency offset 3.5 ppm downfield of water was assigned to amide protons of the protein, and the approach of expressing MTR_asym_ at 3.5 ppm as the apparent APT weighted-imaging has been well established. However, these methods could be affected by background magnetization transfer (MT) effect, direct saturation of bulk water and nuclear overhauser enhancement (NOE). The effect of native MTR_asym_ presumably caused by the solid-phase MTeffect and possible intramolecular and intermolecular nuclear Overhauser effects of aliphatic protons has been known to be difficult to collect^56–58^. We believe that developing a method for analyzing the Z-spectrum that can separate these effects is a future challenge.

In conclusion, the associations of ΔADC_2.5__sADC_27.6_ with different T_d_ values, API SI, and histopathological parameters such as Ki-67 expression and cellularity were found. These results indicate that the T_d_-dependent DWI and CEST parameters are useful for investigating the microstructure of breast cancers. Furthermore, diffusion parameter values, especially sADC, are dependent on T_d_, confirming that this T_d_ dependence is strongly associated with the degree of diffusion hindrance, which increases with the T_d_. These results indicate the importance of reporting T_d_ in future studies.

## Methods

### Cell culture and animal experiments

All animal experiments were performed in accordance with national guidelines and the Regulation on Animal Experimentation at Kyoto University and in compliance with ARRIVE guidelines, and approved by the Kyoto University Animal Care Committee. Two human breast cancer cell lines (MCF-7 and MDA-MB-231) purchased from American Type Culture Collection, Manassas VA, USA were cultured in Dulbecco’s modified Eagle’s medium with 10% fetal bovine serum and 1% penicillin–streptomycin solution at 37 °C with 5% CO_2_.

MCF-7 and MDA-MB-231 cells (1 × 10^6^/μL) were subcutaneously inoculated into the right hindlimbs of 7 and 15 immunodeficient mice (ICR nu/nu 6–8-week-old females; Charles River Laboratories Japan, Yokohama, Japan), respectively. A 17β-estradiol pellet (0.18 mg, 60-day release; Innovative Research of America, Sarasota, FL, USA) was implanted subcutaneously into the neck to promote optimal tumor growth of the estrogen receptor-positive MCF-7 cells. During all procedures, the mice were anesthetized with 2% isoflurane (Wako Pure Chemical Industries, Osaka, Japan). Xenografts were allowed to grow for 7–11 weeks to develop tumors of suitable size.

### MRI of mice

We imaged 22 xenograft mice (7 MCF-7 and 15 MDA-MB-231) on a 7 T MRI scanner (Bruker Biospin, Ettlingen, Germany) using a ^1^H quadrature transmit/receive volume coil. The mice were anesthetized with 1–3% isoflurane in air and were kept still inside the magnet using ear bars and a bite bar connected to a nose cone. Respiration and rectal temperature were continuously monitored using an MR-compatible monitoring system (Model 1025, SA Instruments, Inc., Stony Brook, NY, USA). The rectal temperature was maintained at 34–37 °C.

### Acquisition of DW images and parameters

The 4-segmented multi-slice echo-planar imaging (SE-EPI) acquisition parameters were: TE/TR = 57/2500 ms, 8 averages, matrix size = 100 × 100, field of view = 25 × 25 mm^2^, and slice thickness = 1.5 mm. DWI were obtained using oscillating gradient spin-echo (OGSE)^59^ and classical pulsed gradient spin-echo (PGSE) sequences. Four different T_d_ values were used: 2.5/5 ms in OGSE and 9/27.6 ms in PGSE. The OGSE sequence for T_d_ = 2.5 ms used a trapezoid cosine diffusion gradient at 100 Hz with the following parameters: diffusion gradient length = 20 ms; number of oscillating periods = 2; scan time = 14 min and 40 s.The OGSE sequence for T_d_ = 5 ms used a trapezoid cosine diffusion gradient at 50 Hz with the following parameters: diffusion gradient length = 20 ms; number of oscillating periods = 1; scan time = 22 min and 40 s. PGSE data were acquired with the same parameters (including TE and TR) as the corresponding OGSE data, except diffusion gradient length = 7.2 ms and diffusion separation = 11.4 and 30 ms for T_d_ = 9 and 27.6 ms, respectively. The scan time for PGSE was 22 min and 40 s for each T_d_. We used 11 and 17 b values for T_d_ = 2.5 ms (0, 5, 10, 20, 30, 50, 70, 100, 200, 400, 600 s/mm^2^) and for T_d_ = 5 ms, 9 ms and 27.6 ms (0, 5, 10, 20, 30, 50, 70, 100, 200, 400, 600, 800, 1000, 1500, 2000, 2500, 3000 s/mm^2^), respectively.

The ADC and the shifted ADC (sADC) values were calculated as:

1 $$sADC = \ln S ( {Lb} )/S( {Hb} )/( {Hb - Lb} )$$

where Lb and Hb are a “low key b value” and a “high key b value,” respectively^22^. In this study, sADC is calculated with Lb = 200, Hb = 1500 s/mm^2^; ADC is calculated with the above equation with Lb = 0 (not shifted), Hb = 600 s/mm^2^, therefore, we describe the former as sADC^200–1500^ and the latter as ADC^0–600^.

The change in ADC^0–600^ (ΔADC^0–600^), sADC^200–1500^ (ΔsADC^200–1500^) and ADC between ${\text{ADC}}_{{2.5\,{\text{ms}}}}^{0{-} 600}$ and ${\text{sADC}}_{{27.6\,{\text{ms}}}}^{200{-}1500}$ (ΔADC_2.5__sADC_27.6_) were calculated using the combination of the following ADC/sADC values:

2 $$\Delta ADC_{2.5, 27.6}^{0,600} ( \% ) = (ADC_{t = 2.5\,ms} - ADC_{t = 27.6\,ms} )/ADC_{t = 2.5\,ms} \times 100$$

3 $$\Delta sADC_{9, 27.6}^{200,1500} ( \%) = (sADC_{t = 9\,ms} - sADC_{t = 27.6\,ms} )/sADC_{t = 9\,ms} \times 100$$

4 $$\Delta ADC_{2.5}^{0,600} \_sADC_{27.6}^{200,1500} ( \% ) = (ADC_{t = 2.5\,ms} - sADC_{t = 27.6\,ms} )/ADC_{t = 2.5\,ms} \times 100$$

The rationale for the ΔADC_2.5__sADC_27.6_ was to optimize both effects of the diffusion time and non-Gaussian diffusion.

The signals were also fitted using the IVIM/non-Gaussian diffusion kurtosis model^60,61^

5 $$S ( b) = S ( 0)f exp( { - bD^{*} }) + S_{{0{\text{diff}}}} \exp [ - bADC_{0} + K ( {bADC_{0} })^{2} /6]$$

where S(b) is the noise corrected raw signal intensity at each b value, *f,* the flowing blood fraction (which represents the microvascular flowing volume fraction), D*, the pseudo-diffusion (which represents perfusion-related incoherent microcirculation). S_0 diff_ is the theoretical signal that would be obtained at b = 0 s/mm^2^ taking only the tissue diffusion component into account (corresponding to S(0)[1 − *f*]), ADC_0_ is the virtual ADC that would be obtained when b comes close to 0 s/mm^2^ at this model’s curve, K is kurtosis.

The noise corrected raw signal intensity, S(b), was obtained from the noisy (acquired) raw signal, Sn(b), as S(b) = [|Sn(b)^2^ − NCF|]^1/2^, where NCF (noise floor correction factor) characterizes the “intrinsic” Rician noise contribution observed at low signal intensities within amplitude-reconstructed MR images. The noise floor was estimated from the average image background noise across runs^60^.

The noise corrected signals acquired with each T_d_ at b > 500 s/mm^2^ (free from perfusion-related IVIM effects) were first fitted with Eq. (5) to estimate ADC_0_, K and S_0 diff_.

As a second step, the fitted diffusion signal component, S_diff_(b), (second term of Eq. (5)) was subtracted from the noise corrected raw signal acquired for b < 500 s/mm^2^, and the remaining signal was fitted using the perfusion-related IVIM monoexponential model to obtain estimates of *f* and D* from Eq. (6):

6 $$S ( b) - S_{diff} ( b) = S ( 0) \times f exp ( { - bD^{*} })$$

For the non-Gaussian and IVIM parameters, two T_d_ values (9 ms and 27.6 ms) from PGSE were used.

### Acquisition of CEST images and parameters

CEST slices were obtained at the region of maximum tumor width. CEST images were acquired using the rapid acquisition with relaxation enhancement (RARE) sequence with a single continuous wave saturation pre-pulse. The acquisition parameters were: TR = 5000 ms, effective TE = 12 ms, RARE factor = 16, a centric ordered phase encoding, matrix size = 96 × 96, field of view = 25 × 25 mm^2^, and slice thickness = 2 mm. The saturation parameters were: saturation time = 1 s, saturation RF power = 5.9 μT, saturation offset frequencies with respect to water resonance = 41, range = ± 5 ppm, and 0.25 ppm steps. The acquisition time for each saturation offset was 30 s, and the total acquisition time was 20.5 min. Reference images were also acquired at a saturation frequency of 40 ppm. The water frequency offset caused by B0 inhomogeneity was corrected using the water saturation shift referencing (WASSR) method^62^.

CEST data were analyzed using common asymmetry analysis. The magnetization transfer ratio asymmetry (MTR_asym_) values were calculated as^62,63^:

7 $${\text{MTR}}_{{{\text{asym}}}} ( {{{\Delta \omega }}} ) = \left[ {{\text{S}}\left( { - {{\Delta \omega }}} \right) - {\text{S}}\left( {{{\Delta \omega }}} \right)} \right]/{\text{S}}_{0}$$

where Δω is the shift difference between the irradiation frequency and the water center frequency and S(Δω) and S_0_ are the water intensities after a long presaturation pulse at the offset frequency and without a presaturation pulse, respectively. The APT signal intensity was defined as the MTR_asym_ value at 3.5 ppm (Δω = 3.5 ppm).

### Image and histopathological analysis

The mean values of the diffusion parameters were retrieved for each ROI drawn on DWI images using MATLAB (Mathworks, Natick, MA, USA). Regions of interest (ROIs) were drawn on T2WI as a reference containing the whole tumor region using a freehand tool using ImageJ 1.52a (National Institutes of Health, USA) for CEST, and the ROIs were then copied to the corresponding APT images to obtain their signal intensity. MRI data analysis was performed using code developed in MATLAB (Mathworks, Natick, MA, USA).

The tumor specimens were formalin-fixed and paraffin-embedded, and then sectioned for hematoxylin and eosin (H&E) stains, which were performed in a routine manner. Immunohistochemical staining for Ki-67 (1:100 dilution, Leica Biosystems, NCL-L-Ki67-MM1, Novocostra, Newcastle, UK) and CD31 (1:100 dilution, Cell Signaling Technology, #77699, MA, USA) was performed using the Ventana Discovery XT Autostainer (Tucson, Arizona, USA). With regard to Ki-67 staining, the Ki-67 proliferation index was assessed using only the areas of tissue containing the highest concentrations of cells. The Ki-67 proliferation index was evaluated using the percentage of immunoreactive cells in the area of observation under high power magnification (HPF, × 200). Five values were averaged to determine the final Ki-67 labeling index (Ki-67 LI), which were calculated in a routine manner via microscopic estimation, and the Ki-67_max_ was determined as the maximum of five values. With regard to CD31 staining, the number of all vessels in 10 HPF (× 40), was added after pre-scanning at low magnification (× 10) to choose the area with the impression of the highest vessel profile number (“hot spot”) in the solid area. The result, microvessel density (MVD), was calculated as the mean count of vessels in the HPF viewing range.

The tumor specimens were scanned, converted to whole-slide imaging, and analyzed using automated histology image analysis software (Halo, Indica labs, Corrales, NM, USA). Four areas (cellular, necrotic, muscle, and stromal areas) were separated and quantified using a pattern recognition algorithm. Cellular area was defined as a percentage of the total area. The Ki-67-positive cell map over the whole tumor was also generated using Halo, and the Ki-67-positive area was defined as a percentage of the total area. The Ki-67 positive ratio was defined as the Ki-67-positive area as a percentage of the cellular area. Cell size was defined as the mean diameter of 10 cells observed in a square ROI of size 200 μm × 200 μm. Three square ROIs were drawn in representative viable areas of the tumor, and cell size was averaged over these ROIs.

### Statistical analysis

The estimated parameter values were expressed as mean ± SD. Between-group differences were compared using the Wilcoxon rank sum test. Spearman’s rank correlation coefficients were used to evaluate the correlations between each parameters. *P* values of less than 0.05 were considered statistically significant. All statistical analysis was performed using Medcalc (version 11.3.2.0, Mariakierke, Belgium) and R (version 3.6.1; R Foundation for Statistical Computing, Vienna, Austria) statistical software.

## Supplementary Information

Supplementary Table S1.
