# Supplementary material for: Investigation of breast cancer microstructure and microvasculature from time-dependent DWI and CEST in correlation with histological biomarkers
Source: Sci Rep. 2022 Apr 20;12:6523. doi: 10.1038/s41598-022-10081-7 (PMC9021220; doi:10.1038/s41598-022-10081-7)
Supplement: Supplementary file 1 — Supplementary Table S1. [file 41598_2022_10081_MOESM1_ESM.pdf]

Investigation of breast cancer microstructure and microvasculature from time-dependent DWI and CEST in correlation with histological biomarkers

Yuko Someya[1,*], Mami Iima[1,2], Hirohiko Imai[3], Akihiko Yoshizawa[4], Masako Kataoka[1], Hiroyoshi Isoda[1], Denis Le Bihan[5–7], Yuji Nakamoto[1]

[1] Kyoto University, Graduate school of medicine, Department of Diagnostic Imaging and Nuclear Medicine, Kyoto, 606-8507, Japan
[2] Kyoto University Hospital, Institute for Advancement of Clinical and Translational Science, Department of Clinical Innovative Medicine, Kyoto, 606-8507, Japan
[3] Kyoto University, Graduate School of Informatics, Department of Systems science, Kyoto, 606-8501, Japan
[4] Kyoto University Hospital, Department of Diagnostic Pathology, Kyoto, 606-8507, Japan
[5] NeuroSpin/Joliot, CEA-Saclay Center, Paris-Saclay University, Gif-sur-Yvette, F91191, France
[6] Kyoto University Graduate School of Medicine, Human Brain Research Center, Kyoto, 606-8507, Japan
[7] National Institute for Physiological Sciences, Okazaki, 444-8585, Japan
*someyayuko@kuhp.kyoto-u.ac.jp

Supplementary Information

	MCF−7	MDA−MB−231	P−value
**Histology**	N=7	N=15	
Cell size (μm)	15.2 ± 0.7	12.2 ± 0.3	0.004**
Cellular area (%)	71.8 ± 12.2	51.3 ± 22.7	0.021*
Ki−67 labelling index	58.8 ± 11.5	75.5 ± 3.3	0.003**
Ki−67$_{max}$	63.9 ± 10.0	80.7 ± 3.9	0.001**
Ki67 positive ratio (%)	41.5 ± 12.5	60.2 ± 20.4	0.026*
MVD	23.5 ± 6.8	44.1 ± 15.9	<0.001**

Supplementary Table S1. Histopathological features of MDA−MB−231 and MCF−7 xenograft models. $P < 0.05$*, $P < 0.01$**, considered as statistically significant. MVD; mean vessel density
